# Characterization of Peripheral Immune Cell Subsets in Patients with Acute and Chronic Cerebrovascular Disease: A Case-Control Study

**DOI:** 10.3390/ijms161025433

**Published:** 2015-10-23

**Authors:** Peter Kraft, Christiane Drechsler, Michael K. Schuhmann, Ignaz Gunreben, Christoph Kleinschnitz

**Affiliations:** 1 Department of Neurology, University Hospital Würzburg, 97080 Würzburg, Germany; E-Mails: schuhmann_m@ukw.de (M.K.S.); Gunreben_i@ukw.de (I.G.); kleinschni_c@ukw.de (C.K.); 2 Department of Internal Medicine, University Hospital Würzburg, 97080 Würzburg, Germany; E-Mail: Drechsler_c@ukw.de

**Keywords:** biomarker, immune cells, leukocytes, lymphocytes, monocytes, regulatory T cells, ischemic stroke, chronic cerebrovascular disease, thromboinflammation

## Abstract

Immune cells (IC) play a crucial role in murine stroke pathophysiology. However, data are limited on the role of these cells in ischemic stroke in humans. We therefore aimed to characterize and compare peripheral IC subsets in patients with acute ischemic stroke/transient ischemic attack (AIS/TIA), chronic cerebrovascular disease (CCD) and healthy volunteers (HV). We conducted a case-control study of patients with AIS/TIA (*n* = 116) or CCD (*n* = 117), and HV (*n* = 104) who were enrolled at the University Hospital Würzburg from 2010 to 2013. We determined the expression and quantity of IC subsets in the three study groups and performed correlation analyses with demographic and clinical parameters. The quantity of several IC subsets differed between the AIS/TIA, CCD, and HV groups. Several clinical and demographic variables independently predicted the quantity of IC subsets in patients with AIS/TIA. No significant changes in the quantity of IC subsets occurred within the first three days after AIS/TIA. Overall, these findings strengthen the evidence for a pathophysiologic role of IC in human ischemic stroke and the potential use of IC-based biomarkers for the prediction of stroke risk. A comprehensive description of IC kinetics is crucial to enable the design of targeted treatment strategies.

## 1. Introduction

Peripheral immunodepression is a common observation after acute ischemic stroke [1,2,3] and other acute disorders of the central nervous system (CNS), such as cerebral hemorrhage [4] and spinal cord injury [5]. This phenomenon was first described more than three decades ago [6]. Since then, numerous researchers have tried to delineate the underlying mechanism and its clinical relevance. Today, there is consent that lesions of vulnerable areas within the CNS increase sympathetic activity and subsequently trigger rapid and extensive apoptosis in lymphatic organs via catecholamines and the hypothalamic-pituitary-adrenal axis [7,8]. The adaptive as well as the innate immune systems are involved.

The stroke-induced immunodeficiency [8] has important clinical implications. It is well known that the prognosis of stroke depends on medical complications [9], most of all infections, such as urinary tract infections (up to 24% of stroke patients [10]) or pneumonia (up to 22% of patients [10]). The latter may be due to an enhanced stroke-related aspiration risk in terms of dysphagia, but immunodeficiency may also, in general, increase the vulnerability to post-stroke infections.

Experimental stroke studies in rodents described the pathophysiologic relevance of immune cell subsets [11,12,13,14,15,16] in the development of acute ischemic stroke (AIS). A protective effect of the total absence of lymphocytes [14], lymphopenia [17], or even the temporary depletion of immune cell subsets has been demonstrated [13]. The reduction of immune cells in the cerebral microcirculation lowers thromboinflammation during the acute phase of stroke, and consecutively results in improved cerebral perfusion and protection from stroke [17,18]. As the number of peripheral lymphocytes or their interaction with other cells in the cerebral microcirculation can be modulated pharmacologically [17,19,20], targeting immune cells in the acute phase after ischemic stroke might become a future treatment option and is of high translational relevance. Only recently, two studies analyzing fingolimod in ischemic [21] and hemorrhagic stroke [22] were published. Fu and co-workers recently provided an overview of studies about immune interventions in acute ischemic stroke in humans [23].

While the importance of neuroimmunologic interactions after ischemic stroke—including the role of distinct immune cell subsets—is being increasingly recognized [24,25,26,27,28,29], many questions about the regulation of immune cell subsets are still unanswered and have to be clarified before further translation of novel preclinical treatment strategies into the clinic. Additionally, despite the crucial role of various immune cells in atherosclerotic plaque pathophysiology [30], almost no study has specifically investigated the regulation of immune cells in patients with chronic cerebrovascular disease (CCD) [31].

This case-control study has been conducted to evaluate whether: (i) peripheral immune cell subsets differ between healthy volunteers (HV), patients with acute cerebrovascular disease (AIS/transient ischemic attack [TIA]), and those with CCD; and (ii) to identify demographic and clinical predictors of the numbers of distinct peripheral immune cells in patients with AIS/TIA.

## 2. Results and Discussion

### 2.1. Descriptive Analysis of Patients with Acute Cerebrovascular Disease

Overall, the study included 116 patients with AIS/TIA. Patients had a mean age of 70 ± 12 years, 53% were male and 58% of patients presented with an AIS. Baseline clinical severity, measured using the National Institutes of Health Stroke Scale (NIHSS) and Barthel Index, was 4.8 ± 6.0 and 74 ± 30, respectively. The demographic and clinical characteristics of patients presenting with an AIS or TIA are summarized in Table 1.

**Table 1:** Baseline characteristics of patients with acute ischemic stroke/transient ischemic attack.

Characteristic	Value (*n* = 116)
Age, years	70 ± 12
*Sex, n (%)*	
Male	62 (53)
Female	54 (47)
*Modality, n (%)*	
AIS	67 (58)
TIA	49 (42)
*TOAST criteria, n (%)*	
Cardioembolism	70 (60)
Large-artery atherosclerosis	4 (3)
Small-vessel occlusion	12 (10)
Other determined or undetermined etiology	30 (26)
Thrombolysis, *n* (%)	34 (29)
*Comorbidities, n (%)*	
Hypertension	105 (91)
Diabetes mellitus	41 (35)
Hyperlipidemia	80 (69)
Renal failure	10 (9)
Atrial fibrillation	37 (32)
Persistent foramen ovale	28 (24)
Heart failure	5 (4)
Coronary artery disease	8 (7)
Family history of stroke	11 (9)
Smoking, *n* (%)	18 (16)
*Pretreatment*	
Platelet inhibitor before blood withdrawal, *n* (%)	87 (75)
Anticoagulation before blood withdrawal, *n* (%)	8 (7)
Lipid-lowering drug before blood withdrawal, *n* (%)	36 (31)
*Severity of stroke*	
National Institutes of Health Stroke Scale at admission	4.8 ± 6.0
Barthel Index at admission	74 ± 30
Body mass index, kg/m^2^	27 ± 5
HbA_1c_, mmol/mol	46 ± 13
*Lipid profile, mmol/L*	
Total cholesterol	202 ± 52
Low-density lipoprotein	121 ± 45
High-density lipoprotein	51 ± 15
Triglycerides	157 ± 153
Duration between symptom onset and blood withdrawal, h	14 ± 7

AIS, acute ischemic stroke; HbA_1c_, glycated hemoglobin; TIA, transient ischemic attack; TOAST, Trial of Org 10172 in Acute Stroke Treatment.

### 2.2. Comparison of the Number or Fraction of Distinct Immune Cells in Patients with AIS/TIA, CCD, and HV

**Figure 1:**
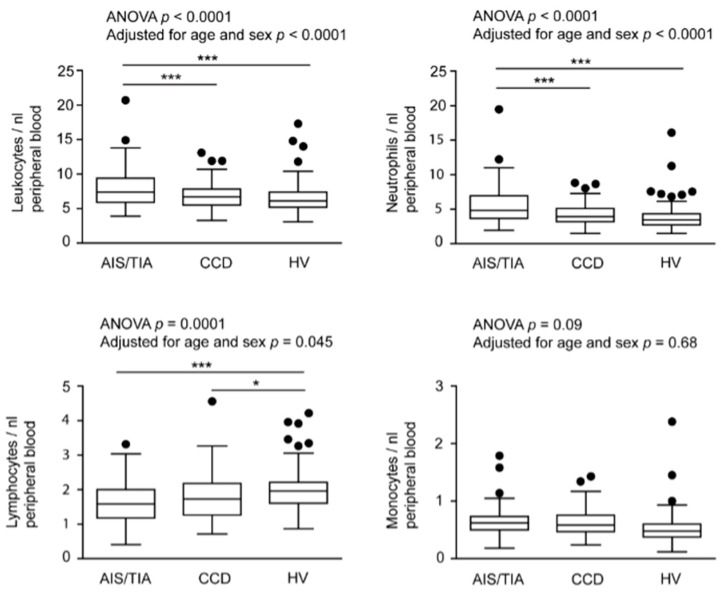

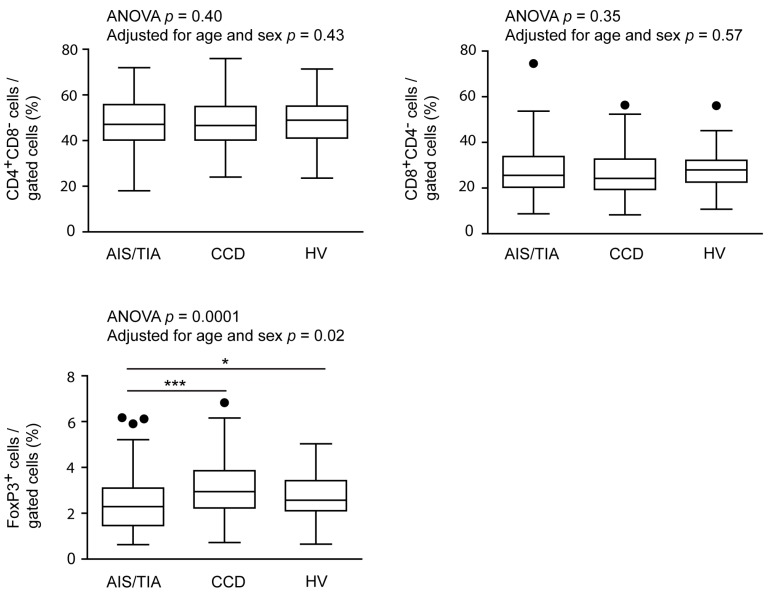
Numbers or fractions of important immune cell subsets in acute ischemic stroke (AIS)/transitory ischemic attack (TIA), chronic cerebrovascular disease (CCD), and healthy volunteers (HV). The number of leukocytes, neutrophils, lymphocytes, monocytes, CD4^+^CD8^−^, CD8^+^CD4^−^, and FoxP3 regulatory T cells (T_reg_) are depicted in box-and-whisker plots indicating the first and third quartiles as well as the 1.5 interquartile range (IQR, Tukey plot). Outliers that lie outside the 1.5 IQR are represented by single dots. The numbers of leukocytes, neutrophils, lymphocytes and FoxP3^+^ T_reg_ differed significantly between the three groups, as determined by analysis of variance with Bonferroni *post* *hoc* test, *** *p* < 0.001, * *p* < 0.05.

The numbers or fractions of immune cell subsets in patients with AIS/TIA and CCD and in HV are shown in Figure 1 for comparison. For the primary analyses of data (without adjustment for confounders, but also adjusted for age and sex), we found significantly higher numbers of leukocytes and neutrophils in patients with AIS/TIA (leukocytes, 7.9 ± 2.7/nL; neutrophils, 5.4 ± 2.6/nL) compared with patients with CCD (leukocytes, 6.8 ± 1.8/nL, *p* < 0.001; neutrophils, 4.2 ± 1.4/nL, *p* < 0.001) and HV (leukocytes, 6.5 ± 2.2/nL, *p* < 0.001; neutrophils, 3.8 ± 1.9/nL, *p* < 0.001). In contrast, lymphocytes were higher in HV (2.0 ± 0.6/nL) compared with patients with CCD 1.8 ± 0.6/nL, *p* < 0.05) and those with AIS/TIA (1.6 ± 0.6/nL, *p* < 0.001). Also FoxP3^+^ regulatory T cells (T_reg_), a subset of lymphocytes, were decreased in patients with AIS/TIA (2.4% ± 1.2%) compared with patients with CCD (3.1% ± 1.2%, *p* < 0.001) and HV (2.8% ± 1.0%, *p* < 0.05). There was no difference in the number of monocytes as well as the fraction of CD4^+^CD8^−^ or CD8^+^CD4^−^ T cells between the groups.

### 2.3. Relationship between the Number or Fraction of Immune Cell Subsets and Key Demographic and Clinical Parameters in Patients with Acute Cerebrovascular Disease

Results from univariate analysis of the association between the number or fraction of immune cells subsets and key demographic and clinical characteristics are summarized in Table 2 and Table 3. Leukocyte (*p* < 0.001) and neutrophil numbers (*p* < 0.001) as well as the fraction of FoxP3^+^ T_reg_ (*p* = 0.02) were higher in patients with AIS compared with patients with TIA. In contrast, the number of monocytes was lower in patients with AIS than in patients with TIA (*p* = 0.02). Older patients showed a lower quantity of lymphocytes (*p* = 0.02) and a smaller fraction of CD4^+^CD8^−^ T cells (*p* = 0.03). Severity of stroke at admission was associated with different immune cell subsets (NIHSS: leukocytes, *p* = 0.05, neutrophils, *p* = 0.007, CD4^+^CD8^−^ cells, *p* = 0.004; Barthel Index: leukocytes, *p* = 0.01, neutrophils, *p* = 0.001). Again, leukocytes (*p* = 0.01) and neutrophils (*p* = 0.003) were associated with thrombolysis. Interestingly, sex and pretreatment with platelet inhibitors did not influence the number or fraction of immune cell subsets.

**Table 2:** Predictors of the absolute number or fraction of immune cells in patients with acute ischemic stroke/transient ischemic attack (univariate analysis; leukocytes, lymphocytes, neutrophils, monocytes).

Immune Cell Subset	Leukocytes/nL (Mean ± SD)	*p* Value	Lymphocytes/nL (Mean ± SD)	*p* Value	Neutrophils/nL (Mean ± SD)	*p* Value	Monocytes/nL (Mean ± SD)	*p* Value
*Sex*								
Male	7.9 ± 2.7	-	1.7 ± 0.5	-	5.4 ± 2.5	-	0.7 ± 0.3	-
Female	7.9 ± 2.6	0.76	1.6 ± 0.7	0.24	5.5 ± 2.7	0.60	0.6 ± 0.2	0.80
*Age, years*								
<55	8.0 ± 3.2	-	2.0 ± 0.6	-	5.1 ± 3.1	-	0.6 ± 0.4	-
55–64	8.0 ± 2.2	-	1.7 ± 0.5	-	5.5 ± 1.9	-	0.6 ± 0.2	-
65–74	7.6 ± 2.2	-	1.7 ± 0.7	-	5.2 ± 1.9	-	0.7 ± 0.3	-
75–84	7.5 ± 2.3	-	1.5 ± 0.5	-	5.3 ± 2.3	-	0.6 ± 0.2	-
>84	9.0 ± 4.5	0.85	1.3 ± 0.6	0.02	6.9 ± 4.7	0.63	0.6 ± 0.2	0.71
*Disease modality*								
AIS	8.6 ± 2.9	-	1.7 ± 0.6	-	6.2 ± 2.8	-	0.6 ± 0.2	-
TIA	6.9 ± 1.9	<0.001	1.6 ± 0.6	0.36	4.4 ± 1.8	<0.001	0.7 ± 0.3	0.02
*Modified TOAST criteria*								
Cardioembolism	8.0 ± 2.9	-	1.6 ± 0.6	-	5.6 ± 2.9	-	0.6 ± 0.2	-
Large-artery atherosclerosis	7.1 ± 2.0	-	1.8 ± 0.4	-	4.6 ± 1.9	-	0.6 ± 0.1	-
Small-vessel occlusion	7.8 ± 2.1	-	1.8 ± 0.5	-	5.0 ± 1.9	-	0.7 ± 0.2	-
Other determined or undetermined etiology	7.7 ± 2.3	0.94	1.5 ± 0.6	0.32	5.4 ± 2.2	0.91	0.6 ± 0.2	0.80
*Duration between symptom onset and blood withdrawal, h*								
<5	7.7 ± 2.2	-	1.6 ± 0.6	-	5.4 ± 2.2	-	0.6 ± 0.2	-
5–12	7.5 ± 2.4	-	1.7 ± 0.6	-	5.0 ± 2.2	-	0.6 ± 0.2	-
12–24	8.4 ± 2.3	0.14	1.3 ± 0.4	0.63	6.3 ± 2.5	0.05	0.7 ± 0.1	0.80
*National Institutes of Health Stroke Scale*								
0–4	7.4 ± 2.2	-	1.7 ± 0.6	-	4.8 ± 2.0	-	0.6 ± 0.2	-
5–9	8.5 ± 2.8	-	1.5 ± 0.4	-	6.2 ± 2.6	-	0.7 ± 0.3	-
10–15	8.8 ± 3.1	-	1.7 ± 0.7	-	6.3 ± 2.9	-	0.7 ± 0.3	-
>15	9.6 ± 4.1	0.05	1.2 ± 0.4	0.06	7.7 ± 4.4	0.007	0.6 ± 0.1	0.88
*Barthel Index*								
0–30	9.7 ± 4.4	-	1.2 ± 0.6	-	7.7 ± 4.7	-	0.6 ± 0.2	-
35–70	8.5 ± 2.7	-	1.6 ± 0.5	-	6.1 ± 2.6	-	0.7 ± 0.3	-
>70	6.9 ± 1.7	0.01	1.8 ± 0.6	0.048	4.3 ± 1.5	0.001	0.6 ± 0.2	0.13
*Thrombolysis*								
Yes	8.6 ± 2.2	-	1.6 ± 0.4	-	6.2 ± 2.3	-	0.7 ± 0.3	-
No	7.6 ± 2.8	0.01	1.7 ± 0.7	0.99	5.1 ± 2.7	0.003	0.6 ± 0.2	0.38
*Platelet inhibitor before blood withdrawal*								
Yes	8.0 ± 2.9	-	1.7 ± 0.6	-	5.6 ± 2.9	-	0.6 ± 0.2	-
No	7.6 ± 2.2	0.60	1.6 ± 0.6	0.42	5.2 ± 2.0	0.83	0.7 ± 0.3	0.81

AIS, acute ischemic stroke; TIA, transient ischemic stroke; TOAST, Trial of Org 10172 in Acute Stroke Treatment.

**Table 3:** Predictors of the absolute number or fraction of immune cells in patients with acute ischemic stroke/transient ischemic attack (univariate analysis; (CD4^+^CD8^−^ T cells, CD8^+^CD4^−^ T cells, FoxP3 T_reg_).

Immune Cell Subset	CD4^+^CD8^−^ Cells/Gated Cells (%) (Mean ± SD)	*p* Value	CD8^+^CD4^−^ Cells/Gated Cells (%) (Mean ± SD)	*p* Value	FoxP3^+^ Cells/Gated Cells (%) (Mean ± SD)	*p* Value
*Sex*						
Male	46.3 ± 11.3	-	29.9 ± 11.6	-	2.4 ± 1.1	-
Female	47.7 ± 11.8	0.51	25.8 ± 9.8	0.04	2.4 ± 1.4	0.36
*Age, years*						
<55	50.1 ± 9.8	-	24.9 ± 9.7	-	2.8 ± 1.3	-
55–64	49.2 ± 11.2	-	28.5 ± 12.2	-	2.3 ± 1.2	-
65–74	50.1 ± 10.1	-	26.7 ± 9.9	-	2.4 ± 1.2	-
75–84	41.6 ± 11.8	-	31.8 ± 11.0	-	2.4 ± 1.3	-
>84	42.5 ± 12.9	0.03	24.9 ± 11.7	0.22	2.2 ± 1.1	0.81
*Disease modality*						
AIS	49.2 ± 10.4	-	26.0 ± 9.6	-	2.7 ± 1.2	-
TIA	45.3 ± 12.1	0.09	29.4 ± 11.7	0.24	2.2 ± 1.2	0.02
*Modified TOAST criteria*						
Cardioembolism	46.0 ± 12.1	-	29.3 ± 11.0	-	2.4 ± 1.3	-
Large-artery atherosclerosis	55.0 ± 12.2	-	21.2 ± 6.7	-	3.3 ± 1.3	-
Small-vessel occlusion	51.1 ± 7.2	-	24.8 ± 8.7	-	2.7 ± 1.2	-
Other determined or undetermined etiology	46.4 ± 11.2	0.27	27.0 ± 12.0	0.35	2.1 ± 1.0	0.23
*Duration between symptom onset and blood withdrawal, h*						
<5	49.4 ± 8.5	-	26.4 ± 8.7	-	2.4 ± 1.0	-
5–12	47.2 ± 11.4	-	27.7 ± 10.1	-	2.5 ± 1.3	-
12–24	49.8 ± 13.8	0.86	18.9 ± 2.1	0.47	2.7 ± 0.2	0.59
*National Institutes of Health Stroke Scale*						
0–4	48.5 ± 10.6	-	26.6 ± 9.5	-	2.5 ± 1.1	-
5–9	48.7 ± 10.6	-	28.6 ± 10.4	-	2.7 ± 1.4	-
10–15	47.5 ± 11.2	-	29.1 ± 7.1	-	2.3 ± 1.3	-
>15	32.2 ± 11.7	0.004	35.2 ± 20.1	0.66	1.4 ± 0.9	0.03
*Barthel Index*						
0–30	43.7 ± 12.0	-	23.8 ± 5.2	-	2.0 ± 1.3	-
35–70	45.6 ± 10.0	-	28.0 ± 11.3	-	2.4 ± 1.2	-
>70	50.3 ± 9.3	0.16	25.7 ± 8.4	0.58	2.7 ± 1.3	0.37
*Thrombolysis*						
Yes	45.4 ± 10.5	-	27.7 ± 13.3	-	2.3 ± 1.4	-
No	47.6 ± 11.9	0.32	28.1 ± 10.0	0.39	2.4 ± 1.1	0.31
*Platelet inhibitor before blood withdrawal*						
Yes	45.6 ± 11.5	-	28.4 ± 10.8	-	2.4 ± 1.2	-
No	49.3 ± 11.2	0.11	27.3 ± 11.5	0.49	2.4 ± 1.2	0.97

AIS, acute ischemic stroke; TIA, transient ischemic stroke; TOAST, Trial of Org 10172 in Acute Stroke Treatment.

Multivariate analysis (adjusted for age and sex) (Table 4, Table 5, Table 6 and Table 7) identified disease modality (AIS *vs.* TIA) as an independent predictor of leukocyte (*p* = 0.006), neutrophil (*p* = 0.005), and monocyte count (*p* = 0.04). Sex was only associated with the fraction of CD8^+^CD4^−^ T cells (*p* = 0.03). Age only accounted for CD4^+^CD8^−^ T cell regulation (*p* = 0.012). NIHSS scores were independent predictors of the CD4^+^CD8^−^ as well as the CD8^+^CD4^−^ fractions (*p* = 0.02 and 0.03, respectively). We found no independent predictor of FoxP3 T_reg_. In addition, none of the measured variables (leukocytes, *p* = 0.48; neutrophils, *p* = 0.54; lymphocytes, *p* = 0.81; monocytes, *p* = 0.81; CD4^+^CD8^−^ T cells, *p* = 0.65; CD8^+^CD4^−^ T cells, *p* = 0.65; FoxP3 T_reg_, *p* = 0.95) were influenced by the time of blood withdrawal (Days 0, 1, and 3; data not shown).

**Table 4:** Predictors of absolute numbers of leukocytes and lymphocytes in patients with acute ischemic stroke/transient ischemic attack (multivariate analysis).

Immune Cell Subset		Leukocytes			Lymphocytes		
		Coefficient	95% CI	*p* Value	Coefficient	95% CI	*p* Value
Sex	Male	Reference	-	-	Reference	-	-
	Female	−0.4 ± 0.9	−1.1 to 1.0	0.93	0.0 ± 0.1	−0.2 to 0.3	0.71
Age, years		-	-	0.74	-	-	0.003
<55		Reference	-	-	Reference	-	-
55–64		−0.5 ± 0.9	−2.2 to 1.3	-	−0.3 ± 0.2	−0.7 to 0.1	-
65–74		−0.4 ± 0.8	−2.0 to 1.3	-	−0.4 ± 0.2	−0.7 to 0.0	-
75–84		−0.7 ± 0.9	−2.4 to 1.0	-	−0.5 ± 0.2	−0.9 to −0.1	-
>84		0.5 ± 1.1	−1.7 to 2.6	-	−0.6 ± 0.2	−1.1 to −0.2	-
Disease modality (TIA *vs.* AIS)		1.5 ± 0.5	0.4 to 2.6	0.006	−0.1 ± 0.1	−0.3 to 0.2	0.66
National Institutes of Health Stroke Scale		-	-	0.11	-	-	0.052
0–4		Reference	-	-	Reference	-	-
5–9		0.8 ± 0.8	−0.7 to 2.3	-	−0.3 ± 0.2	−0.6 to 0.1	-
10–15		0.8 ± 0.9	−0.9 to 2.6	-	−0.1 ± 0.2	−0.5 to 0.3	-
>15		1.3 ± 1.0	−0.8 to 3.3	-	−0.5 ± 0.2	−1.0 to −0.1	-
Thrombolysis		0.1 ± 0.7	−1.3 to 1.4	0.94	0.1 ± 0.2	−0.20 to 0.4	0.53
Use of platelet inhibitor before blood taking		0.5 ± 0.5	−0.6 to 1.6	0.34	0.2 ± 0.1	−0.1 to 0.4	0.17

AIS, acute ischemic stroke; CI, confidence interval; TIA, transient ischemic stroke.

**Table 5:** Predictors of absolute numbers of neutrophils and monocytes in patients with acute ischemic stroke/transient ischemic attack (multivariate analysis).

Immune Cell Subset		Neutrophils			Monocytes		
		Coefficient	95% CI	*p* Value	Coefficient	95% CI	*p* Value
Sex	Male	Reference	-	-	Reference	-	-
	Female	−0.0 ± 0.5	−1.0 to 0.9	0.98	−0.0 ± 0.1	−0.1 to 0.1	0.53
Age, years		-	-	0.25	-	-	0.53
<55		Reference	-	-	Reference	-	-
55–64		−0.1 ± 0.8	−1.8 to 1.5	-	−0.0 ± 0.1	−0.2 to 0.1	-
65–74		0.1 ± 0.8	−1.5 to 1.6	-	0.0 ± 0.1	−0.1 to 0.2	-
75–84		−0.1 ± 0.8	−1.7 to 1.5	-	−0.0 ± 0.1	−0.2 to 0.1	-
>84		1.2 ± 1.0	−0.8 to 3.2	-	−0.1 ± 0.1	−0.3 to 0.1	-
Disease modality (TIA *vs.* AIS)		1.5 ± 0.5	0.5 to 2.5	0.005	0.1 ± 0.1	0.0 to 0.2	0.04
National Institutes of Health Stroke Scale		-	-	0.031	-	-	0.72
0–4		Reference	-	-	Reference	-	-
5–9		1.0 ± 0.7	−0.4 to 2.5	-	0.0 ± 0.1	−0.1 to 0.2	-
10–15		1.0 ± 0.8	−0.7 to 2.6	-	0.0 ± 0.1	−0.1 to 0.2	-
>15		1.8 ± 1.0	−0.2 to 3.7	-	0.0 ± 0.1	−0.2 to 0.2	-
Thrombolysis		0.0 ± 0.6	−1.2 to 1.3	0.96	−0.0 ± 0.1	−0.1 to 0.1	0.88
Use of platelet inhibitor before blood taking		2.4 ± 1.2	0.1 to 4.7	0.46	−0.0 ± 0.1	−0.1 to 0.1	0.58

AIS, acute ischemic stroke; CI, confidence interval; TIA, transient ischemic stroke.

**Table 6:** Predictors of fractions of CD4^+^CD8^−^ and CD8^+^CD4^−^ T cells in patients with acute ischemic stroke/transient ischemic attack (multivariate analysis).

Immune Cell Subset		CD4^+^CD8^−^ Cells			CD8^+^CD4^−^ Cells		
		Coefficient	95% CI	*p* Value	Coefficient	95% CI	*p* Value
Sex	Male	Reference	-	-	Reference	-	-
	Female	2.8 ± 2.1	−1.3 to 6.9	0.18	−4.7 ± 2.1	−8.8 to −0.5	0.03
Age, years		-	-	0.012	-	-	0.63
<55		Reference	-	-	Reference	-	-
55–64		−0.5 ± 3.6	−7.7 to 6.7	-	3.8 ± 3.6	−3.4 to 11.0	-
65–74		−1.5 ± 3.4	−8.2 to 5.3	-	3.4 ± 3.4	−3.4 to 10.2	-
75–84		−8.7 ± 3.5	−15.7 to −1.6	-	6.9 ± 3.6	−0.2 to 14.0	-
>84		−5.0 ± 4.4	−13.7 to 3.7	-	−0.6 ± 4.4	−9.3 to 8.2	-
Disease modality (TIA *vs.* AIS)		−2.7 ± 2.2	−7.1 to 1.7	0.22	2.5 ± 2.2	−1.9 to 6.9	0.26
National Institutes of Health Stroke Scale		-	-	0.02	-	-	0.03
0–4		Reference	-	-	Reference	-	-
5–9		1.5 ± 3.1	−4.6 to 7.6	-	3.3 ± 3.1	−2.9 to 9.4	-
10–15		−0.0 ± 3.8	−7.5 to 7.5	-	2.1 ± 3.8	−5.5 to 9.7	-
>15		−12.9 ± 4.2	−21.2 to −4.7	-	11.2 ± 4.2	2.8 to 19.5	-
Thrombolysis		−1.1 ± 2.7	−6.4 to 4.2	0.67	−0.3 ± 2.7	−8.6 to 2.1	0.24
Use of platelet inhibitor before blood taking		−1.9 ± 2.2	−6.3 to 2.4	0.39	−0.7 ± 2.2	−5.1 to 3.7	0.77

AIS, acute ischemic stroke; CI, confidence interval; TIA, transient ischemic stroke.

**Table 7:** Predictors of fraction of FoxP3 T_reg_ in patients with acute ischemic stroke/transient ischemic attack (multivariate analysis).

Immune Cell Subset		FoxP3^+^ Cells		
		Coefficient	95% CI	*p* Value
Sex	Male	Reference	-	-
	Female	−0.0 ± 0.2	−0.5 to 0.5	0.95
Age, years		-	-	0.57
<55		Reference	-	-
55–64		−0.3 ± 0.4	−1.1 to 0.5	-
65–74		−0.4 ± 0.4	−1.2 to 0.4	-
75–84		−0.3 ± 0.4	−1.2 to 0.5	-
>84		−0.2 ± 0.5	−1.2 to 0.8	-
Disease modality (TIA *vs.* AIS)		−0.4 ± 0.3	−0.9 to 0.2	0.16
National Institutes of Health Stroke Scale		-	-	0.13
0–4		Reference	-	-
5–9		0.3 ± 0.4	−0.4 to 0.1	-
10–15		−0.1 ± 0.4	−0.9 to 0.8	-
>15		−1.0 ± 0.5	−2.0 to −0.1	-
Thrombolysis		0.1 ± 0.3	−0.5 to 0.7	0.80
Use of platelet inhibitor before blood taking		0.1 ± 0.3	−0.4 to 0.6	0.69

AIS, acute ischemic stroke; CI, confidence interval; TIA, transient ischemic stroke; FoxP3 T_reg_, regulatory T cells.

### 2.4. Discussion

In this case-control study, we analyzed peripheral immune responses in different cerebrovascular disease settings and showed that the number or fraction of distinct immune cell subsets is differentially regulated between patients with AIS/TIA and CCD, compared with HV. Moreover, within the AIS/TIA group, several clinical (e.g., NIHSS, Barthel index, thrombolysis or not, AIS or TIA) or demographic (age, sex) parameters predicted the number or fraction of immune cells even after adjustment for age and sex.

Most of our findings in patients with AIS/TIA are consistent with the results of previously published studies regarding ischemic stroke [1,2,32,33,34,35]. However, in contrast to other observations [24,36], we found no change in the number of monocytes during the observation period until day 3. Of note, a low fraction of CD4^+^CD8^−^ T cells and a high percentage of CD8^+^CD4^−^ T cells were independently associated with high clinical severity of patients with AIS/TIA at admission. This observation can be confirmed by a study showing that subjects with a high fraction of CD8^+^ cells often have comorbidities that include insulin resistance and an increased risk of cardiovascular events [36]. Therefore, despite evidence for reduced cytotoxic function of CD8^+^ T cells in AIS [26], it can be hypothesized that especially CD8^+^ cells have detrimental properties in cardiovascular disease. FoxP3 T_reg_ were associated with clinical severity in univariate analysis but not after adjustment for age and sex.

Very importantly, other diseases of the CNS—such as like aneurysmal subarachnoid hemorrhage [4] or acute cerebral hemorrhage [25]—are also associated with changes in peripheral immune cell homeostasis and distribution, showing that peripheral immune cell modulation is an unspecific response to various acute CNS diseases [7,8]. Nevertheless, kinetics of immune cell regulation might be different between various CNS diseases. In contrast to our results in ischemic stroke, Shi *et al.* [25] reported an increase in T_reg_ over time in patients with intracerebral hemorrhage and Sarrafzadeh *et al.* [4] found an increase in CD4^+^ and CD8^+^ T cells in a subpopulation of patients in the first days after aneurysmal subarachnoid hemorrhage.

Despite clear evidence that inflammatory mechanisms and immune cells play an important part in the pathophysiology of atherosclerosis—including plaque progression and instability [31,37]—to the best of our knowledge, this report is the first description of the detailed regulation of immune cell subsets in CCD. The numbers of leukocytes, neutrophils, and lymphocytes in patients with CCD lie between those seen for patients with AIS/TIA and HV, pointing towards a hypothetical sequence of disease from healthy persons to chronic cerebrovascular atherosclerotic lesions (extracranial and/or intracranial) and finally AIS. Very interestingly, FoxP3^+^ T_reg_ were even higher in patients with CCD compared with those with AIS/TIA, suggesting a pathophysiologic role of T_reg_ in CCD. Atherosclerosis is currently understood as a systemic disease that might also be influenced by pro- and anti-inflammatory cytokines. A recent report suggested that the level of detrimental cytokines could be decreased by physical exercise [38]. Further studies are needed to better understand the underlying pathophysiology.

As immune cells are not only biomarkers after ischemic stroke, but also potential therapeutic targets [23], a detailed characterization of their regulation is absolutely necessary for elaborating the best treatment strategy and also for improving the translation of promising preclinical agents into the clinic. We identified several variables that independently predicted the number or fraction of various immune cell subsets (AIS *vs.* TIA, age, sex, NIHSS). It seems that the number of immune cells depends on various non-modifiable clinical and demographic variables, making it difficult to develop universal treatment strategies.

There are several limitations to this study that should be considered. First, it should be remembered that the potential for reverse causation as a result of blood withdrawal following a cerebrovascular event cannot be disregarded. Accordingly, the current study describes the magnitude and significance of associations between immune cell subsets and demographic/clinical parameters without attributing causality. Further prospective studies are required to formally elucidate causality. Second, all patients were required to provide informed consent before participating in the study. However, this may have resulted in patients who have suffered a severe stroke and/or aphasia being underrepresented in this study because neurological deficits related to their condition may have prevented them from being capable of providing informed consent. Third, it was not possible to completely rule out a non-vascular origin for symptoms in 42% of the TIA patient population, meaning that the possibility of the aforementioned factors influencing the regulation of immune cell subsets remains.

## 3. Experimental Section

### 3.1. Data Collection

Patients with acute cerebrovascular disease (AIS/TIA) and CCD were included in this study, while control subjects were HV from the local population. All study participants were required to meet the following inclusion criteria: for patients presenting with an AIS (*i.e.*, an acute ischemic lesion on brain imaging) and TIA (no acute ischemic lesion on brain imaging), blood samples must have been drawn within 24 hours of symptom onset; in the CCD group, patients must have presented with extracranial and/or intracranial stenosis of the large cerebral arteries with (*n* = 66) or without (*n* = 51) a history of AIS or TIA; and for the control HV subjects, aged ≥50 years with no history of stroke, myocardial infarction, or peripheral arterial disease. Patients with AIS, TIA or CCD were excluded from the study if they presented with intracerebral hemorrhage, were aged <18 years, had a known plasmatic coagulation disorder, or a detailed medical history indicated the presence of platelet dysfunction.

Study participants were consecutively recruited between September 2010 and January 2013 from inpatients diagnosed with TIA or AIS in the Stroke Unit, outpatients presenting with CCD, and the HV population who responded to recruitment advertisements in the Neurology Department, University Hospital of Würzburg, Germany. The study protocol was approved by the ethics committee of the Medical Faculty of the University of Würzburg, Germany (reference number 65/2010) and written informed consent was provided by all participants. In total, 337 patients were eligible to participate in the study, including 116 patients with AIS or TIA, 117 patients with CCD, and 104 HV. Patient treatment and care remained at physician discretion and was not affected by participation in this study.

An adapted version of the TOAST (Trial of Org 10172 in Acute Stroke Treatment) criteria [39] was applied to patients who presented with acute cerebrovascular disease (AIS or TIA): (1) cardioembolism; (2) large-artery atherosclerosis; (3) small-vessel occlusion; or (4) other determined or undetermined etiology. On patient admission, the interval between symptom onset and blood withdrawal, platelet inhibitor pretreatment, and acute stroke therapy modality (thrombolysis *vs.* no thrombolysis) were recorded, as well as NIHSS [40] and Barthel Index scores [41].

### 3.2. Blood Collection and Measurements

Blood samples were drawn from an antecubital vein using a 21-gauge butterfly needle between 08.00 and 12.00 h on Days 0, 1, and 3 in patients with acute cerebrovascular disease. Blood samples were only drawn once in patients with CCD and HV. Pre-analytic preparations for blood collection were carried out according to specific standard operating procedures and only non-hemolyzed blood samples were analyzed. Differential hematology—including the absolute number of leukocytes, lymphocytes, neutrophils and monocytes—has been analyzed at the Division of Laboratory Medicine of the University Hospital Würzburg. Flow cytometric analysis of the fractions of CD4^+^CD8^−^, CD8^+^CD4^−^, and FoxP3^+^ T_reg_ was performed using peripheral blood mononuclear cells (PBMCs), with density gradient centrifugation used to isolate PBMCs from peripheral blood. Cells were analyzed on a BD FACSCalibur flow cytometer (BD Biosciences, Heidelberg, Germany). The following primary antibodies were used: FoxP3-APC (Cat. no.: 17-4776-42; eBiosciences, Frankfurt, Germany), CD4^−^ FITC (Cat. no.: 347413, BD Biosciences, Heidelberg, Germany), and CD8^−^ PE (Cat. no.: 555635; BD Biosciences, Heidelberg, Germany). The respective isotype controls were purchased from BD Biosciences. The gating strategy is illustrated in Supplementary Figure S1.

### 3.3. Statistical Analysis

Continuous variables are presented as mean ± standard deviation or median with interquartile range, as appropriate. Categorical variables are expressed as percentages. Analysis of variance (ANOVA) and chi-square tests were used to investigate the association between the absolute number or fraction of immune cell subsets and demographic and clinical characteristics (age, sex, neurologic scales, disease modality (TIA or AIS), TOAST criteria, duration between symptom onset and blood withdrawal, NIHSS score, Barthel Index score, treatment modality (intravenous thrombolysis or not), and treatment with platelet inhibitors in the days before blood withdrawal) and *p* values derived, as appropriate. Coefficients and corresponding 95% confidence intervals for potential predictors of the numbers of distinct immune cells were estimated using a linear regression model that included all variables without collinearity in a multivariate model that was adjusted for age and sex. Immune cell subsets were compared between the different patient groups (inpatients with AIS/TIA, outpatients with CCD, or HV), and distributions analyzed using the Kolmogorov-Smirnov test. It was assumed that the immune cell numbers were normally distributed and the groups were compared using ANOVA with a Bonferroni *post-hoc* test. These comparisons were additionally adjusted for age and sex. All reported *p* values are derived from two-sided tests, with a *p* value <0.05 considered to be statistically significant. Analyses were performed using SPSS Version 21 and SAS software version 9.1 (SAS Institute Inc., Cary, NC, USA).

## 4. Conclusions

Changes in peripheral immune cell numbers are a well-known signature after ischemic stroke. At the same time, immune cell subsets play major roles in the pathophysiology of murine ischemic stroke and might also become future targets of novel therapeutic approaches in humans. We provide here an overview of the regulation of distinct immune cell subsets after AIS/TIA in comparison with CCD and HV. The description of independent predictors of immune cells raises new questions, which might be valuable for the understanding of pathophysiologic mechanisms, and could finally help to enable focused treatment strategies.

## Supplementary Materials

Supplementary materials can be found at http://www.mdpi.com/1422-0067/16/10/25433/s1.
