# Supplementary material for: Characterization of Peripheral Immune Cell Subsets in Patients with Acute and Chronic Cerebrovascular Disease: A Case-Control Study
Source: Int J Mol Sci. 2015 Oct 23;16(10):25433–49. doi: 10.3390/ijms161025433 (PMC4632808; doi:10.3390/ijms161025433)
Supplement: Supplementary file 1 [file ijms-16-25433-s001.pdf]

Supplementary Information

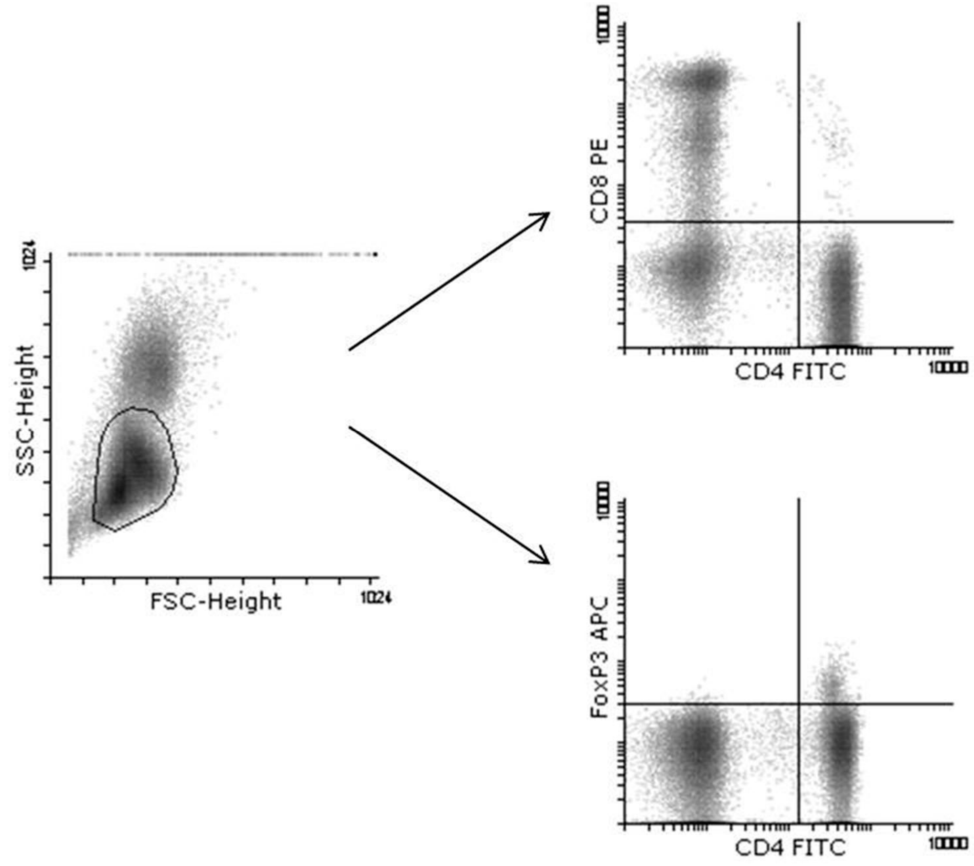

Figure S1. Gating strategy for flow cytometric analysis of the fractions of $CD4^{+}CD8^{-}$, $CD8^{+}CD4^{-}$ and $CD4^{+}FoxP3^{+}$ cells. The surrounded cell population (left graph) indicates the viable lymphocytes. APC, allophycocyanin; PE, phycoerythrin; FITC, fluorescein isothiocyanate; FSC, forward scatter; SSC side scatter.
